# A Novel Machine Learning-Based Methodology for Tool Wear Prediction Using Acoustic Emission Signals

**DOI:** 10.3390/s21175984

**Published:** 2021-09-06

**Authors:** Juan Luis Ferrando Chacón, Telmo Fernández de Barrena, Ander García, Mikel Sáez de Buruaga, Xabier Badiola, Javier Vicente

**Affiliations:** 1 Vicomtech Foundation, Basque Research and Technology Alliance (BRTA), Mikeletegi 57, 20009 Donostia-San Sebastian, Spain; tfernandez@vicomtech.org (T.F.d.B.); agarcia@vicomtech.org (A.G.); 2 Faculty of Engineering, Mondragon University, 20500 Mondragon, Spain; msaez@mondragon.edu (M.S.d.B.); xbadiola@mondragon.edu (X.B.); jvicentet@mondragon.edu (J.V.)

**Keywords:** tool wear, machine learning, wavelet packet transform, acoustic emission, condition monitoring, predictive maintenance

## Abstract

There is an increasing trend in the industry of knowing in real-time the condition of their assets. In particular, tool wear is a critical aspect, which requires real-time monitoring to reduce costs and scrap in machining processes. Traditionally, for the purpose of predicting tool wear conditions in machining, mathematical models have been developed to extract the information from the signal of sensors attached to the machines. To reduce the complexity of developing physical models, where an in-depth knowledge of the system being modelled is required, the current trend is to use machine-learning (ML) models based on data from the tool wear. The acoustic emission (AE) technique has been widely used to capture data from and understand the real-time condition of industrial assets such as cutting tools. However, AE signal interpretation and processing is rather complex. One of the most common features extracted from AE signals to predict the tool wear is the counts parameter, defined as the number of times that the amplitude of the signal exceeds a predefined threshold. A recurrent problem of this feature is to define the adequate threshold to obtain consistent wear prediction. Additionally, AE signal bandwidth is rather wide, and the selection of the optimum frequencies band for feature extraction has been pointed out as critical and complex by many authors. To overcome these problems, this paper proposes a methodology that applies multi-threshold count feature extraction at multiresolution level using wavelet packet transform, which extracts a redundant and non-optimal feature map from the AE signal. Next, recursive feature elimination is performed to reduce and optimize the vast number of predicting features generated in the previous step, and random forests regression provides the estimated tool wear. The methodology presented was tested using data captured when turning 19NiMoCr6 steel under pre-established cutting conditions. The results obtained were compared with several ML algorithms such as k-nearest neighbors, support vector machines, artificial neural networks and decision trees. Experimental results show that the proposed method can reduce the predicted root mean squared error by 36.53%.

## 1. Introduction

Tool wear is a complex phenomenon due to its high variability. It is an important feature for machining processes monitoring because it affects the surface roughness, dimensional accuracy and the cutting process itself, as the amount of energy needed to remove the metal directly depends on the degree of tool wear [1].

Tool wear is generated as a result of chemical, thermal and mechanical interactions between the tool and workpiece materials. These interactions are the cause of the two main types of tool wear that can define the end of tool-life: flank wear $\left(V_{b}\right)$ and crater wear [2]. The effectiveness of the process is commonly linked to the degree of flank wear. Therefore, this variable is taken as a wear indicator for the industry. It has been widely studied that the increase in flank wear makes an increase in the cutting forces [3], which directly drives to an increase in the power consumption of the machine tool. Furthermore, flank wear not only affects the forces and power consumption, but also modifies the contact dynamics between the tool and the material to be cut. As described in the review published in [4], acoustic emissions (AE) are widely employed for monitoring flank wear in turning processes.

AE is a phenomenon whereby transient elastic waves are generated by, e.g., plastic deformation, crack propagation, erosion, corrosion, impact, or leakage [5]. Applications of AE for non-destructive testing are found in numerous industries, including refineries, pipelines, power generation (nuclear or other), aircraft, offshore oil platforms, paper mills and structures (bridges, cranes, etc.) [6]. In the last three decades AE has centered the attention of researchers for its sensitivity in monitoring interacting surfaces in the field of tribology such as the processes present in machining. Liang et al. [7] described the following possible sources of AE during cutting processes:

(a) Plastic deformation during the cutting process in the workpiece;
(b) Plastic deformation in the chip;
(c) Frictional contact between the tool flank face and the workpiece resulting in flank wear;
(d) Frictional contact between the tool rake face and the chip resulting in crater wear;
(e) Collisions between chip and tool;
(f) Chip breakage;
(g) Tool chipping and fracture.

Frequency content of AE signals generated in friction processes covers a broad frequency range, between 50 kHz and 1 MHz [8]. Since the distance and the existence of joints between the AE source and sensor are critical due to the high attenuation of the signals, the preferred mounting location of the AE sensor is the cutting tool or workpiece. However, in real applications, the sensor must be mounted in the tool holder due to the ephemeral nature of the cutting tool and workpiece.

In the literature, several research works have been carried out to diagnose the condition of the tool in turning processes based on AE signals. An extensive review study that presents the research activities using the AE signals to monitor and control various machining processes is presented in [9]. Jose et al. [10] studied the impact of wear on force and AE signals in turning processes of D2 steel concluding that AE parameters increased proportionally with tool wear. One of the most widespread features used in diagnosis and prognosis using AE signals is the counts, defined as the number of times the amplitude exceeds a pre-set voltage (threshold level) in a given time. This feature has been used and investigated extensively, for example in bearing and gear fault diagnosis applications. However, the use of this feature in machining applications is very limited. The main barrier of using this feature is the fact that determining the threshold levels has been at the discretion of the researcher and in most cases, and the values are probably selected depending on intuition and/or experience on the particular test-rig or machine [11]. The threshold is often set above the noise level that allows to distinguishing AE events from the background noise. However, the noise level can vary according to the operational condition of the machine within the machining process. It has been pointed out that the threshold level would have to be calibrated for each specific machining condition and the selection of a threshold level for the AE count rate is usually arbitrary [12]. Das et al. [13] stated that, as the threshold is usually subjectively selected, variation is observed among different testing conditions and researchers. Due to such variation, the derived damage indicators might not be sufficiently accurate for engineers to make optimal real-time data driven decisions. Kwak et al. [14] applied a counts parameter to monitor a grinding process. In this case, the authors set the threshold as 20 mV to extract the counts feature, which was determined by a preliminary experiment. However, in this research as in many others [15,16] the authors did not mention the specific methodology used to select the optimum threshold level to extract this feature.

Wavelet transform (WT) is a well-known technique where useful information from the signal can be extracted at different frequency scales. It is frequently applied to AE signals as a pre-processor to decompose the signal in frequency bands before feature extraction is performed and has been successfully applied to AE signals in different scenarios [17]. This process is known as multiresolution analysis (MA). Benkedjouh et al. [18] presented a new intelligent method for tool wear condition monitoring based on continuous wavelet transform (CWT) and blind source separation (BSS) techniques. They concluded that the proposed CWT-BSS method can effectively reflect the performance degradation of cutting tools for the milling process. Hong et al. [19] presented a novel tool wear monitoring method for determining the state of a micro-end mill using wavelet packet transforms and Fisher’s linear discriminant. The recognition results were compared with those of an energy-based monitoring technique and found that the method proposed could determine the tool state more accurately for both normal wear and premature failure of micro-end mills. Leng et al. [20] showed that the RMS value of the AE signals and the energy of the wavelet packet are correlated with the tool wear in drilling process. However, in many cases, the number of features extracted at MA was redundant and non-optimal [21], limiting the capability of predictors to estimate the tool wear. 

To reduce the error incurred by machine learning (ML) regression models, feature selection techniques are an efficient tool to select the meaningful information from predicting variables. These techniques can be structured into three categories: filter, wrapper and embedded methods, depending on how they combine the feature selection procedure with the construction of the learning model [22]. Recursive feature elimination (RFE) is a popular embedded method, and it is much more robust to data over-fitting than other feature selection techniques. Deshpande et al. [23] successfully applied RFE based on logistic regression as a feature selection tool with AE sensor and ML frameworks to classify different wear categories simulated with a customized pin-on-disc tribometer. 

The theory that predicts condition and wear based on signals captured in real-time is known as prognostics and health management (PHM). These methods can be split into three categories: physic-based, classical model-based and data-driven prognostics. Classical model-based prognostics refer to approaches based on mathematical models of system behavior derived from physical laws or probability distribution. For example, model-based prognostics include methods based on Wiener and gamma processes, hidden Markov models (HMMs), Kalman filters, and particle filters. Examples of the application of model-based prognostics for tool wear using AE can be found in [24,25,26,27]. One of the drawbacks of using model-based prognostics, as well as physic-based models, is that an in-depth knowledge of the physics involved in the process is required. On the other hand, data-based models use approaches that develop predictive models based on ML algorithms such as autoregressive models, artificial neural networks (ANN) or support vector machines (SVM) and random forest (RF). The main benefit of these types of models is that an in-depth knowledge of the physics involved in the process is not required. To date, several ML algorithms have been applied successfully to AE signals for tool wear prediction such as SVM and ANN. Li et al. [28] compared the performance of several ML methods such as ANN and random forests (RF) to predict tool wear in a milling process. They concluded that RF generates more accurate predictions than other ML methods. However, in this research as well as other publications reviewed by the authors, the predicting variables used as input to the ML predictors were the traditional statistical features, such as RMS, maximum value, standard deviation, etc. from the complete frequency spectrum, which may not be optimal and could limit the accuracy of the models.

The main objective of this work is to present a novel methodology for tool wear prediction based on AE signals, proposing four steps with associated ML algorithms and parameter configuration guidelines. This novel technique combines wavelet packets for MA, and feature extraction of both RMS and counts at different thresholds at different WPT nodes. The proposed methodology also presents RFE to remove redundant and highly correlated variables generated by multiresolution and multi-threshold feature extraction, to reduce the prediction error. One of the advantages of the proposed method is the reduction of the complexity of setting the optimum threshold level for the count parameter identified in the literature. Several ML methods are compared to obtain the lowest error for flank tool wear prediction. The results obtained using this novel method are compared with traditional feature extraction methods and ML regressors. The optimum segmentation is investigated to predict tool flank wear. In addition, a brief analysis on the difference of the AE signal patterns in the time-domain and frequency-domain is presented in both low and high tool flank wear condition.

## 2. Materials and Methods

### 2.1. Methodology Proposed

**Figure 1:**
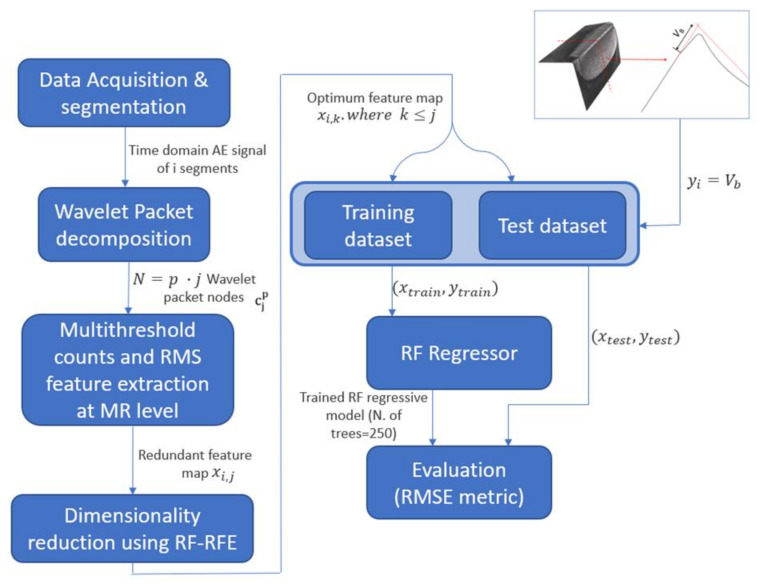
Graphical description of the proposed methodology.

This section presents the methodology for tool wear prediction using WPT, multiresolution (MR) feature extraction feature extraction, RFE and RF. The methodology is depicted in Figure 1 and described as follows:

Step 1—Wavelet packet decomposition: MA is applied to the AE signal using WPT to extract the features presented in step 2 at a different frequency resolution.

Step 2—MR feature extraction: From each WPT node, numerous features $x_{i,j}$, where i is the number of observations and j the number of features, are extracted. These are counts parameters at different thresholds and RMS. This feature extraction technique is presented in Section 2.1.2.

Step 3—Dimensionality reduction using RF-RFE: Since the volume of predictors $x_{i,j}$ extracted from step 2 is extremely high and redundant, by using RF-RFE algorithm, correlated and redundant features are eliminated reducing the number of predictors $x_{i,k}$, where k≤j. This algorithm decreases the prediction time of ML algorithms and reduces the prediction error.

Step 4—Flank tool wear prediction using RF regressive model: The features selected from the WPT nodes using RF-RFE $\left(x_{i,k}\right)$ and the measured tool flank wear $y_{i}$ are used to train and test a RF regression algorithm. The methodology is evaluated using root mean squared error (RMSE) metric. The results are compared with ANN, SVM, K-nearest neighbors (KNN) and decision trees (DT).

The description of the processing tools for each step is presented in the following sections.

#### 2.1.1. Wavelet Packet Decomposition

The WT is a mathematical tool, which transforms sequential data in the time axis to the spectral data in both time and frequency [29]. In contrast with sinusoids, wavelets are localized in both the time and frequency domains, so wavelet signal processing is suitable for non-stationary signals, whose spectral content changes over time. 

The CWT is a decomposition of an input function using scaled and translated versions of a wavelet function known as mother wavelet. Mathematically the wavelet coefficients are extracted using the function below:

(1) $$WT_{\psi }\left\{x\right\}\left(a,b\right)=x,\psi_{a,b}=\int x\left(t\right)\psi_{a,b}\left(t\right)d\left(t\right)\;$$

where $\psi_{a,b}$ is the mother wavelet.

The DWT is the analogous mathematical tool of CWT for discrete functions. It is used for digital signal analysis. The DWT consists of identifying the parameters $c_{ke}$ $d_{j,k},\;k\in \mathbb{N},\;j\in \mathbb{N}$ of the equation:

(2) $$f\left(t\right)=\overset{\infty }{\underset{k=-\infty }{\sum }}c_{k}\phi \left(t-k\right)+\overset{\infty }{\underset{k=-\infty }{\sum }}\overset{\infty }{\underset{j=0}{\sum }}d_{j,k}\psi (2^{j}t-k)\;$$

where $\phi \left(t\right)$ and $\psi \left(t\right)$ are the function known, respectively, as father wavelet and mother wavelet. The father wavelet is in fact a scaling function that depends on the mother wavelet. The $\phi \left(t\right)$ and $\psi \left(t\right)$ can be calculated as sequences $h=\left\{h_{n}\right\}\;n\in \mathbb{Z}$ and $g=\left\{g_{n}\right\}\;n\in \mathbb{Z}$:

(3) $$h_{n}=\langle \psi_{1,n},\psi_{0,0}\rangle {}_{e}\psi \left(t\right)=\sqrt{2}\underset{n\mathbb{Z}\in }{\sum }h_{n}\phi \left(2t-n\right)$$

and

(4) $$g_{n}=\langle \phi_{1,n},\phi_{0,0}\rangle {}_{e}\phi \left(t\right)=\sqrt{2}\underset{n\mathbb{Z}\in }{\sum }h_{n}\phi \left(2t-n\right)$$

These two sequences are the base of the DWT.

The common procedure of applying the DWT is through a filter bank where the filter determined by the coefficients $h=\left\{h_{n}\right\}\;n\in \mathbb{Z}$ corresponds to a high-pass filter and $g=\left\{g_{n}\right\}\;n\in \mathbb{Z}$ corresponds to a low-pass filter.

The filters h and g are linear operators that can be applied to a digital input signal x as a convolution:

(5) $$c\left(n\right)=\underset{k}{\sum }g\left(k\right)x\left(n-k\right)=g*x$$

and

(6) $$d\left(n\right)=\underset{k}{\sum }h\left(k\right)x\left(n-k\right)=h*x$$

The signal c(n) is known as approximation and d(n) as detail.

It is possible to repeat the filters shown in Equations (6) and (7) generating a cascade of high-pass and low-pass filters. This tree is known as a filter bank. However, this decomposition filter may not be precise enough to obtain necessary information from the signal. A more detailed frequency resolution can be obtained by implementing WPT to the signal. In a similar way to the DWT, the WPT tree is obtained by:

(7) $$c_{j+1}^{2p}\left[m\right]=\sqrt{2}\overset{\infty }{\underset{n=-\infty }{\sum }}g\left[n-2m\right]c_{j}^{p}\left[n\right]$$

(8) $$c_{j+1}^{2p+1}\left[m\right]=\sqrt{2}\overset{\infty }{\underset{n=-\infty }{\sum }}h\left[n-2m\right]c_{j}^{p}\left[n\right]$$

where j is the depth of the node and p indexes the nodes in the same depth, every $c_{j}^{p}$ with p even is associated to approximations and every $c_{j}^{p}$ with p odd is associated to details. The WPT is a generalization of the wavelet decomposition that offers further decomposition. Consequently, it provides better frequency resolution for the decomposition of the signal [30].

**Figure 2:**
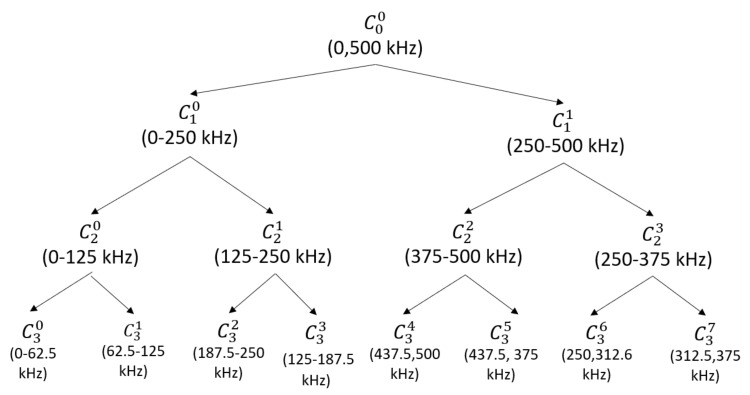
WPT levels, nodes and corresponding frequency bands.

The number decomposition levels investigated in this study was 3 to have a compromise between frequency resolution and computation efficiency since increasing the number of levels increases exponentially the computation required. A total of 15 wavelet nodes were extracted. The WPT decomposition level, nodes and corresponding frequency ranges proposed are presented in Figure 2. As the sampling frequency (fs) to capture the AE signal was set at 1 MHz, the bandwidth used to decompose the AE signal using WPT was 500 kHz, which met the Nyquist frequency (fs/2). It is expected that for most cases the decomposition proposed could achieve optimum results, nevertheless, for other applications the decomposition levels can be expanded or reduced for specific computing and accuracy constraints.

#### 2.1.2. Multiresolution Feature Extraction

This process consists of the extraction of features at MR level and using different thresholds for count feature extraction. Counts were calculated as the number of times that the amplitude of the signal exceeds a predefined threshold. For this particular dataset, 10 different count features were calculated at different thresholds (i.e., 1, 2, 5, 10, 20, 50, 100, 200, 500, 1000 mV) in order to have enough resolution and range (from 1 mV to 1 V) since the AE events and background noise at different WPT nodes had different amplitudes. It was expected that this distribution of thresholds could be adequate for most cases, however, this value list could be expanded according to the AE signal levels. Furthermore, RMS values were extracted at each WPT node. The RMS of a signal $X_{n}$ is defined as follows:

(9) $$RMS=\sqrt{\frac{1}{n}\sum_{i=1}^{n}{\left(x_{i}\right)}^{2}}$$

where n is the number of samples of the signal and $x_{i}$ the samples values. In total 165 features were extracted at MR level, 150 count features (15 WPT and 10 different thresholds) and 15 RMS features; 1 per WPT node. Additionally, traditional features from the complete time domain signal were calculated, i.e., crest factor (CF), peak value, RMS, kurtosis and counts extracted at an arbitrary threshold, set at 0.2 V, but these features were only used as a comparison for the proposed methodology. The nomenclature used for the features extracted was Feature_WPTnode. For the counts feature, since it was extracted using several thresholds the nomenclature was C_Threshold _WPTnode. For example, for the feature counts with threshold 0.01 V, in the WPT level 2, and node 7, the nomenclature was Counts_0.01_$C_{2}^{7}.$

#### 2.1.3. RF-RFE Dimensionality Reduction

**Figure 3:**
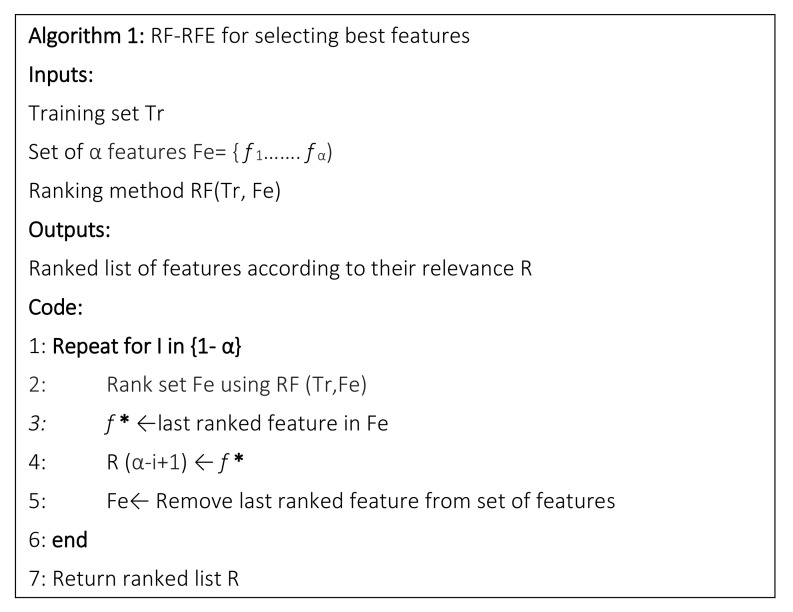
Pseudo-code of recursive feature elimination process.

High-dimensional data often contain a lot of redundant and irrelevant information, which reduce the efficiency of the predictive models for classification [31]. In order to build efficient and effective predictive models, it is, therefore, necessary to select a subset with the most discriminative features. In this study, the redundant and highly correlated set of features generated at MA level in the previous step were reduced and optimized using the RFE. The RFE technique implements a backward selection of the AE features by ranking their importance to an initial model using all the predictors and ranks features according to its importance [32]. It is a greedy optimization procedure used to find the superlative performing subset of features. RFE requires a model to estimate the ranking of the input features. Compared with other models such as SVM or logistic regression, used in [23], RF has been proven to be more effective, which can use fewer features to get higher classification accuracy [33]. Thus, RFE based on RF (RF–RFE) is a feature selection method that combines RF to estimate the error for each recursive feature deleted, and RFE whose process is explained as below (see Figure 3).

#### 2.1.4. Random Forests Regression

**Figure 4:**
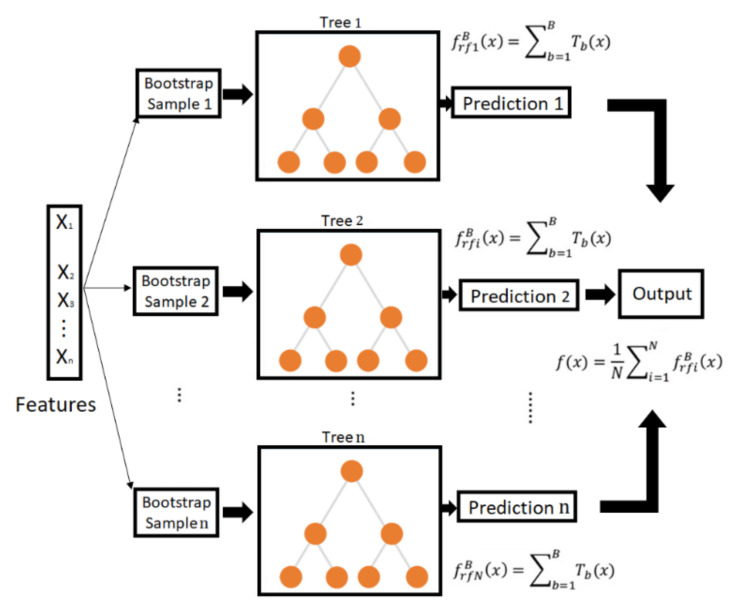
Schematic of tool wear prediction using RF.

The framework of predicting tool flank wear using an RF is illustrated in Figure 4. The optimum feature map estimated by the RF-RFE algorithm is split into train and testing. Then these two arrays of data are used as an input for a RF regressor. The RF algorithm was initially developed by Breinmann [34]; it is an ensemble method that constructs a forest decision trees from bootstrap samples of a training dataset. When coming to predictions, each tree predicts a class, and the class with the most votes is the one selected by the model. A comprehensive tutorial on RFs can be found in [35].

In a normal decision tree, all training data and all features are used to train the model. However, to minimize correlations between different trees in random forests, two main techniques are used:

Bagging: Decision trees are sensitive to the data they are trained with. For that reason, many sub-samples with replacements are created from the training dataset, and each of them is used to train a decision tree. Given a dataset, $D=\left\{\left(x_{1},y_{1}\right),\;\left(x_{2},y_{2}\right),\ldots .,\;\left(x_{n},y_{n}\right)\right\}$, bagging generates B new training datasets Di of size N by sampling from the original training dataset D with replacement. The number of regression trees B is a parameter specified by the users. Bagging reduces variance and avoids overfitting. In this research, a RF is constructed using B = 250 regression trees.

Use of different features: In a normal decision tree, when a feature must be selected in a node, all features are considered. However, in random forest, just one among a random subset of features is selected. 

One of the major advantages of the random forest classifier over other decision tree methods is that the fully-grown trees are not pruned [36]. Breiman [37] suggests that as the number of trees increases, the generalization error always converges even without pruning the trees. The number of features used at each node to generate a tree and the number of trees to be grown are two user-defined parameters that can be optimized for each case. Finally, the metric used to evaluate the performance of the methodology is RMSE, which is defined as follows:

(10) $$RMSE=\sqrt{\frac{1}{n}\overset{n}{\underset{i=1}{\sum }}{\left(\hat{y_{i}}-y_{i}\right)}^{2}}$$

where ${\hat{y}}_{l}$ is the predicted value, $y_{i}$ the observed values and n the observed sample size.

### 2.2. Experimental Procedure

**Figure 5:**
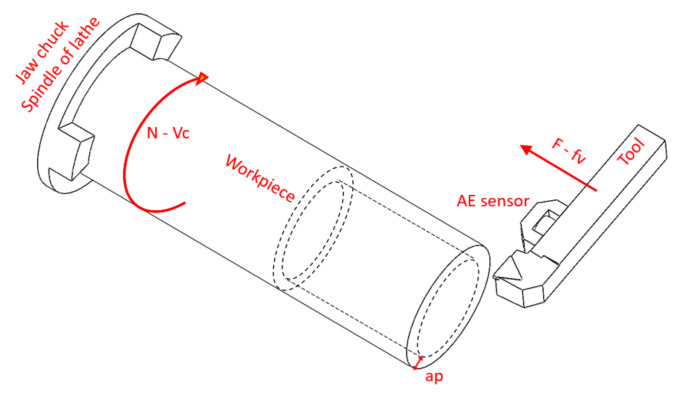
Schematic representation of the turning process and location of AE sensor.

The experimental procedure was based on a conventional 3D turning operation, as is the most common machining procedure for wear testing, which was performed in a Danumerik CNC lathe. In this case, a cylindrical bar of length 250 mm and diameter 60 mm was clamped to the chuck of the spindle, which gave the rotational movement to the workpiece. The machining was performed by a longitudinal movement of the tool towards the workpiece (see Figure 5). The rotation speed of the workpiece (N) and the feed movement of the tool (F) are dependent on the cutting conditions selected for the tests. All the trials were performed in dry conditions at fixed cutting conditions, with a cutting speed (Vc) of 200 m/min, a feed rate (fv) of 0.1 mm/rev and a depth of cut (ap) of 2 mm.

The material employed for the tests was 19NiMoCr6 steel. Concerning the cutting tools, P25 grade uncoated inserts were employed, reference Widia TPUN160308TTM. These were clamped to a Widia CTGPL2020K16 tool holder, which gave an effective rake and clearance angle of 5° and 6°, respectively, with a positioning angle of 90°.

**Figure 6:**
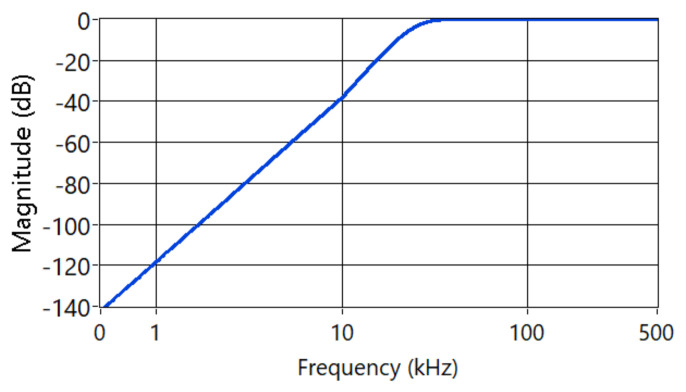
Magnitude response of the Butterworth filter applied to AE signals.

The AE were recorded with a Kistler 8152B sensor coupled with a Type 5125B conditioning system. This was magnetically attached to the tool holder, as shown in Figure 5. The fs was set at 1 MHz, using a National Instrument cDAQ-9171 with an analog input module NI-9223. The AE signal was filtered using a Butterworth high-pass filter with cutting frequency $f_{c}=20\;$ kHzand order 4 to reduce the mechanical noise of the process [38]. The magnitude response of the filter is shown in Figure 6.

**Figure 7:**
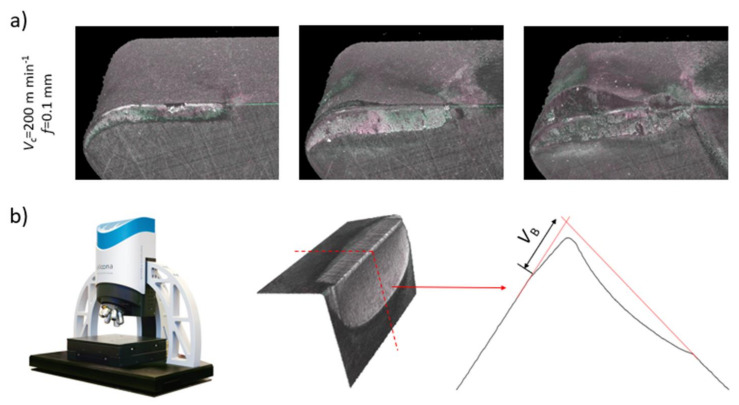
Wear measurement strategy with Alicona Infinite Focus G4: (**a**) 3D topology of worn tool and (**b**) extracted 2D profile of worn tool.

The cutting procedure was as follows:

(1) Machining of a predefined length of the workpiece, commonly 1/3 of the available length (70 mm).
(2) Cleaning of the tool insert to remove adhered material and to enable a correct measurement of tool wear.
(3) Tool wear measurement using an Alicona Infinite Focus G4 profilometer. This profilometer permits the 3D measurement of the wear in the flank (flank wear).
(4) Restart the process (1)–(3) until wear in the flank face exceeds a value of 300 µm.
(5) Figure 7a shows 3D data sets of the evolution of tool wear of one of the repetitions, obtained with the Alicona Infinite Focus G4. To establish flank wear $\left(V_{b}\right)$ from each captured 3D geometry of the worn tool, the profile of the mid plane of the contact section was extracted. The localization and an example of a profile are shown in Figure 7b. The measured wear mode, $V_{b}$, is specified in the profile.

Due to the high variability that wear has in machining operations, the number of repetitions carried out was 12 per tool, to develop predictive models with a high degree of confidence. A total of 5 tools were degraded until the flank wear reached 250 µm.

## 3. Results

**Figure 8:**
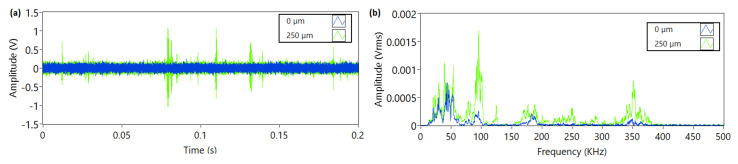
(**a**) Time-domain and (**b**) frequency-domain signals captured from tool with $V_{b}=0$ µm and $V_{b}=250$ µm.

Figure 8a displays the time-domain AE signal associated with 0 and 250 µm wear (maximum investigated). It clearly shows a remarkable difference. While 0 µm has no sign of high amplitude AE shots associated with discrete AE events the plot of 250 µm wear shows numerous discrete AE events of different amplitude above the continuous AE levels in the entire signal. This change in the pattern is associated with tool wear, where several AE sources could be present, such as increased frictional contact between the part and the tool or plastic deformation. Contrary, continuous AE signal remains at similar levels. Figure 8b shows the frequency-domain signal obtained applying FFT to the previous signals. The frequency pattern of no wear condition shows a low-frequency content, with most of the energy in the range 20–60 kHz, which is associated with a continuous AE signal. There is also relatively high energy in the ranges 80–110 kHz, 170–190 kHz and low energy around 350 kHz. The spectrum obtained in a high-wear condition (250 µm) shows similar amplitude at lower frequencies (20–60 kHz). However, the energy in the band 80–110 kHz is highly increased as well as the band around 350 kHz. There is also an increased energy content in the band 200–300 kHz. The energy above 400 kHz is extremely low in both cases. From the analysis of both time-domain and frequency-domain signal we can extract that the pattern changes significantly with varying wear level and is mainly associated with discrete high-frequency AE events.

**Figure 9:**
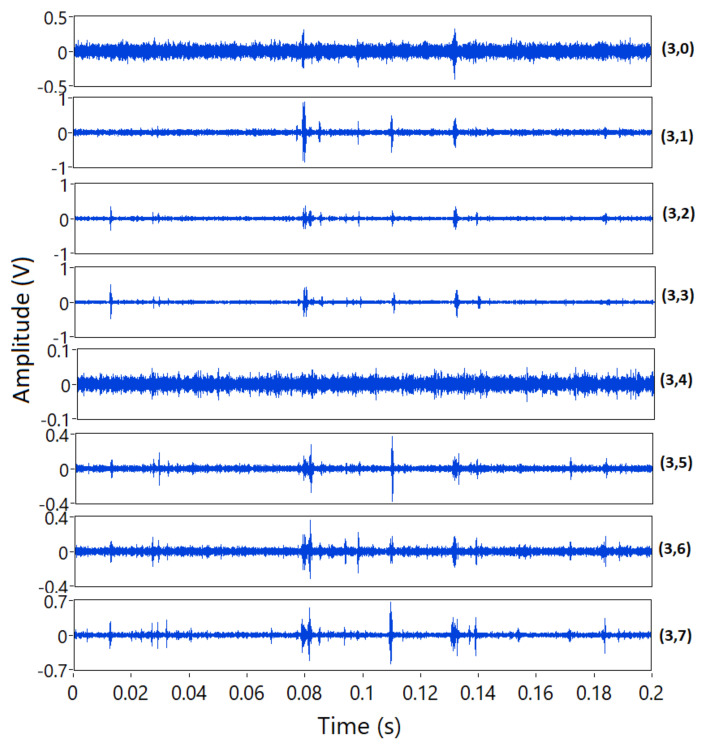
The eight nodes of the AE signal extracted from WPT 3-level decomposition.

Figure 9 displays the decomposition of the previous signal (250 µm wear) using WPT in three levels $\left(C_{3}^{0}-C_{3}^{7}\right)$ using Daubechies_11 wavelet. It shows that the discrete AE events have different characteristic frequencies. While some of the events are evident in a particular band, some are not obvious in others. Therefore, extracting the counts parameter at MR level can provide an advantage by detecting the different events regardless of frequency content.

**Figure 10:**
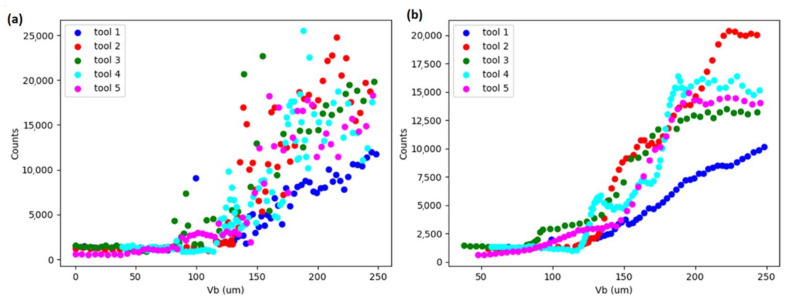
Scatter plot of counts versus flank wear (**a**) original and (**b**) applying moving average filter.

Figure 10 displays a scatter of counts extracted using 50 mV threshold at WPT node $C_{3}^{7}$, which corresponds to 312.5–375 kHz, against flank wear, calculated for 1 s AE signal segments (637 segments in total) for the five tools investigated in this study. It shows very low dispersion up to approximately 90 µm wear. In this range, there is also no variation of counts against wear, which indicates low sensitivity for flank tool wear. From 90 µm until the maximum wear investigated, 250 µm, there is an evident increasing trend of counts with high dispersion. Figure 10b shows the same variables but applying moving average (k = 10), reducing the dispersion to observe the trend clearly.

**Figure 11:**
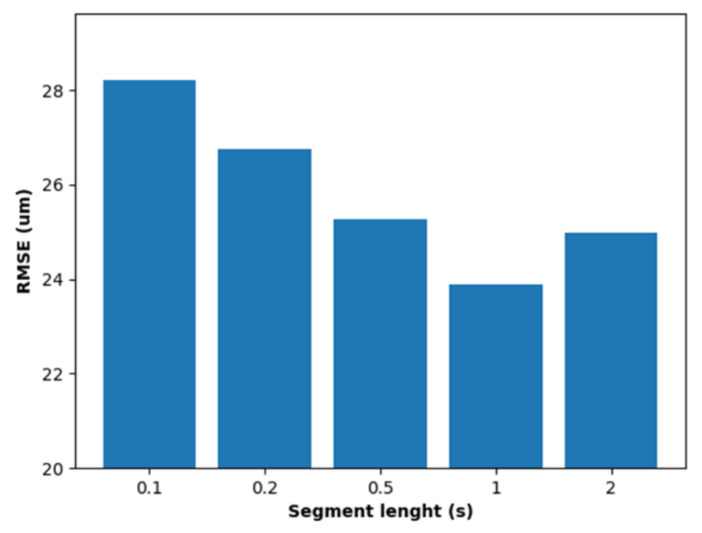
RMSE in predicting tool flank wear vs segment length.

The optimum segment size was evaluated for 5 different segment durations, ranging from 0.1 to 2 s. Figure 11 shows the results obtained which shows that the RMSE error decreases from 0.1 to 1 s, increasing afterwards using 2 s segments, with 1 s being the optimum segment size.

To evaluate the performance of the methodology proposed, the accuracy of the ML models were assessed introducing three different sets of predicting variables using 1 s segment length, determined as the optimal. Firstly, traditional AE features from the complete AE spectrum (WPT was not applied), i.e., kurtosis, peak, RMS, crest factor and counts. These features served as a baseline comparison of the proposed method since they have been traditionally used to predict tool wear by other authors [28,39]. Secondly, all 165 features extracted at MR level were utilized to predict flank wear. Finally, the variables identified by the RFE-RF algorithm as optimum predictors from all 165 features extracted were used. Table 1 shows a summary of the features used in the ML models to predict flank tool wear for the three cases investigated. Since the method is non-deterministic, the optimum features were evaluated 50 times using RFE-RF, obtaining that the most repeated set of features were RMS_$C_{2}^{2}$, Counts_0.05_$C_{3}^{3}$, Counts_0.05_$C_{3}^{7}$, Counts_0.1_$C_{3}^{1}$. Thus, the number of predictors was reduced from 165 to 4. It should be noted that none of the features from the WPT level 0 (complete spectrum) were selected as optimal predictor by the RFE-RF. This points out the importance of using WPT to increase the accuracy of prediction.

**Figure 12:**
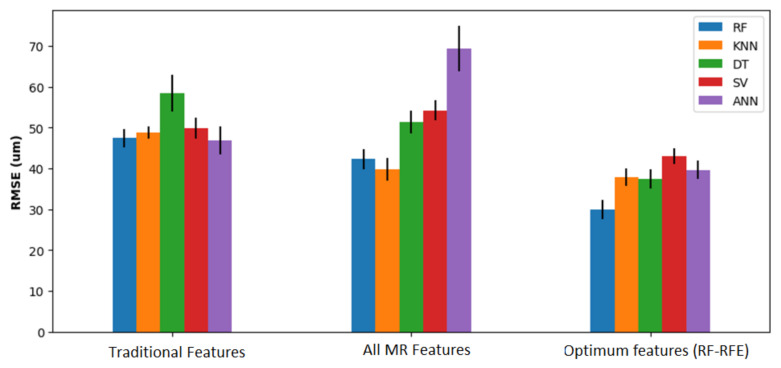
RMSE error in flank tool wear prediction using different ML models and different set of predicting features (lower is better).

In total 673 observations were used to train and test the ML models. The data of two out of the five tools tested were used for testing, while the observations of the other three tools were used for training. The models were trained and tested 10 times varying randomly the data used for testing and training, keeping the ratio of data acquired from three tools for training and two for testing and the results presented are the mean of the individual results. The choice of hyper-parameters for the different regressors investigated was chosen based on an extensive grid search. Figure 12 shows the RMSE obtained using the different ML models and features. Generally, the highest RMSE was obtained using traditional features as predictors, followed by all features extracted from MR feature extraction. The lowest RMSE obtained was 29.91 µm by using RF (number of trees 250) for the MR feature extraction using the optimum features selected by RFE-RF method. Using the traditional features, the lowest RMSE obtained was 47.13 µm by using the RF regressor (N = 250). Thus, using the proposed methodology, the RMSE was reduced by 17.22 µm, which corresponds to a 36.53% drop. In addition, the use of RF-RFE reduced the RMSE significantly, from 39.12 µm using KNN regressor with all MR features to 29.91 µm using optimal features, which corresponds to a 23.54% drop. These results demonstrate the importance of using both WPT prior to feature extraction and RF-RFE for selecting optimal features.

**Figure 13:**
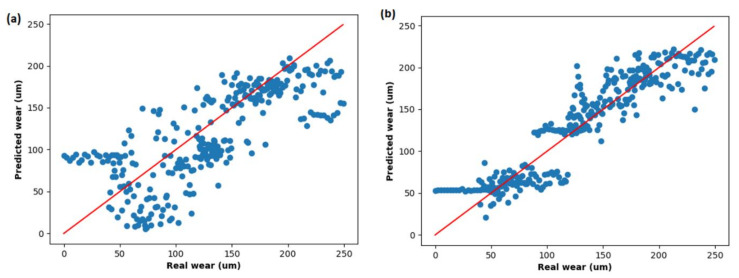
Predicted wear using RF versus real wear in using both (**a**) traditional features and (**b**) MR feature extraction (RF-RFE) technique.

Figure 13 shows a scatter plot of predicted vs real wear using both traditional features and MR feature extraction with RFE-RF feature selection method. It clearly shows a significant reduction in the deviation from the red line (RMSE 0 µm) in the latter case. It is worth noting that the RMSE error in low wear condition is higher (predicts nearly constant wear of 50 µm) which is attributed to a flat relationship up to approximately 90 µm displaying the scatter plot of Counts_0.05_$C_{3}^{7}$ vs. wear shown in Figure 10.

## 4. Conclusions

In this paper, the prediction of flank tool wear in a turning process was conducted by using a novel methodology for AE signals. The performance of the novel method was measured comparing the results with traditional AE features. Several regressive ML algorithms, including RF, SVM, ANN, KNN and DT were investigated to obtain the lowest RMSE predicting flank tool wear. The experimental results have shown that by using the proposed methodology, the RMSE is reduced from 47.13 to 29.91 µm, which corresponds to a 36.53% drop.

One of the most common features extracted from AE signals to predict the tool wear is the counts parameter, defined as the number of times that the amplitude of the signal exceeds a predefined threshold. In the literature, the threshold for extracting this feature is usually set arbitrarily, and no mention of the methodology is detailed. Thus, its value may not be optimum in most cases. Applying the methodology proposed in this paper, an in-depth knowledge of the technique is not required by the operator to set the threshold, as it relays on the RF-RFE technique to select the optimum threshold and frequency band to predict tool wear. 

According to the results obtained using RF-RFE technique, among all the features extracted, the RMS in the band 250–500 kHz, the counts parameter in the band 62.5–125, 125–187.5 and 312.5–375 kHz are the best predictors of tool flank wear. The authors emphasize the use of counts as a reliable feature to predict tool wear, as three out of four optimum features ranked by RF-RFE algorithm correspond to this feature and very few publications were found by the authors that make use of this feature in machining applications, in contrast to other applications, such as bearing or gear health diagnosis. The optimum frequencies and thresholds may vary in different scenarios according to different parameters such as sensor sensitivity, noise, sensor installation, couplant type, distance from sensor to the source of AE, etc. For this reason, the application of RF-RFE for each scenario is expected to be critical. On modern CNC machines, various workpieces are processed with different tools and cutting modes. As it is expected to obtain different AE patterns such as frequency content and amplitude for each process, the methodology proposed must be applied to all of them independently, so the algorithm can learn optimum features, frequencies and patterns of each process.

The sensitivity of AE for predicting flank tool wear is worse at wears lower than 90 µm. The main variation of the AE signal with increasing wear is the increase in number of high amplitude and high frequency discrete events rather than continuous AE levels. Finally, the segmentation size for this particular use case was investigated and 1 s length provides the best results for predicting tool flank wear.

Future extension of this work may include the evaluation of the methodology proposed using variable cutting conditions such as cutting speed and feed per revolution to investigate whether it still outperforms traditional procedures under varying operational conditions. In addition, the presented methodology has been developed and validated to monitor tool flank wear. However, crater wear, chipping and intense self-oscillation are also critical indicators of machining process. The application of the proposed methodology will be evaluated for predicting these parameters in future investigations. Finally, the use of further parameters extracted wavelet nodes, along with RMS and counts used in this research, will be evaluated in future extensions of this work.

## Figures and Tables

**Table 1:** Features selected for each feature cluster.

Traditional Features	MR Extracted Features	Selected Features by RFE_RF
RMS, CF, Peak_ Kurtosis, Counts_0.2	All (165 in total)	RMS_$C_{2}^{2}$, Counts_0.05_$C_{3}^{3}$, Counts_0.05_$C_{3}^{7}$, Counts_0.1_$C_{3}^{1}$

## Data Availability

Data sharing not applicable.
